# Supplementary material for: Neutrophil Extracellular Traps Correlate with Tumor Necrosis and Size in Human Malignant Melanoma Metastases
Source: Biology (Basel). 2023 Jun 6;12(6):822. doi: 10.3390/biology12060822 (PMC10295294; doi:10.3390/biology12060822)
Supplement: Supplementary file 1 [file biology-12-00822-s001.zip › Figure S1.pdf]

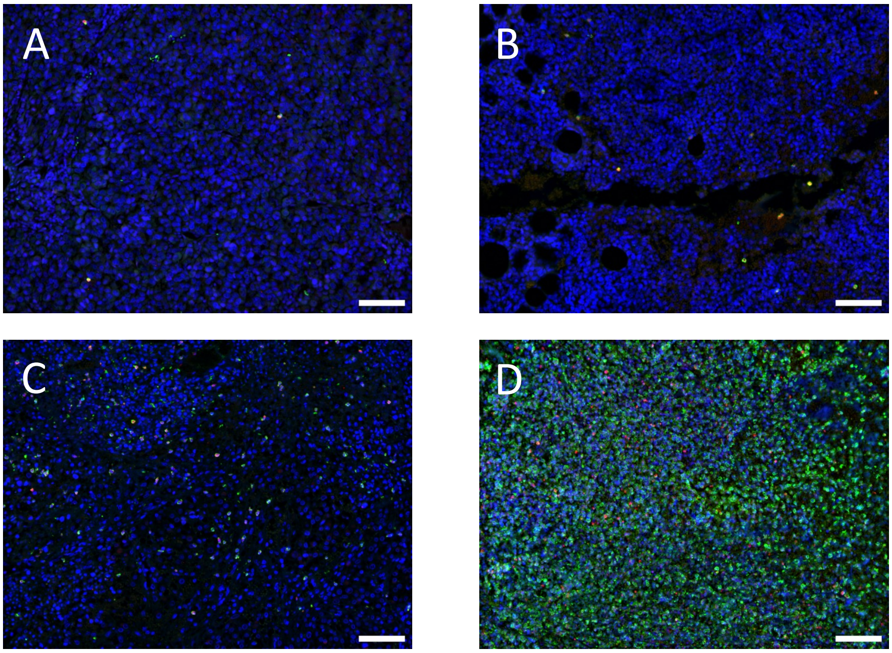

Figure S1. Examples of different infiltration levels and corresponding classification. **A** only sporadic: N0, NET0 **B** more abundant neutrophils, with nuclear but no extracellular H3Cit: N1, NET0 **C** widely distributed neutrophils, partly showing NETs: N2, NET2 **D** dense neutrophil infiltration with NETs: N3, NET3. Scale bars = 100µm.
