# Supplementary material for: Neutrophil Extracellular Traps Correlate with Tumor Necrosis and Size in Human Malignant Melanoma Metastases
Source: Biology (Basel). 2023 Jun 6;12(6):822. doi: 10.3390/biology12060822 (PMC10295294; doi:10.3390/biology12060822)
Supplement: Supplementary file 1 [file biology-12-00822-s001.zip › Figure S2.pdf]

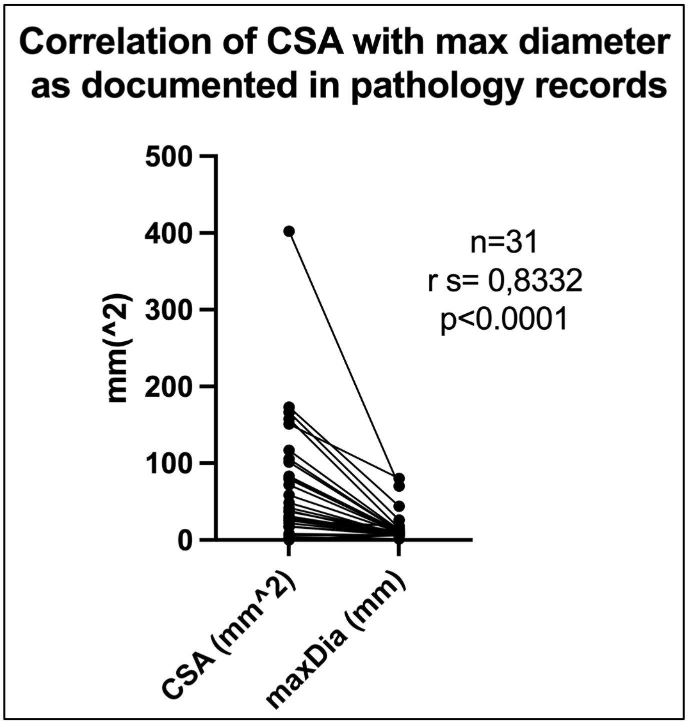

Figure S2. Validation CSA correlation with max diameters of removed metastases in clinical records. r s = Spearman correlation coefficient.
