# Supplementary material for: Neutrophil Extracellular Traps Correlate with Tumor Necrosis and Size in Human Malignant Melanoma Metastases
Source: Biology (Basel). 2023 Jun 6;12(6):822. doi: 10.3390/biology12060822 (PMC10295294; doi:10.3390/biology12060822)
Supplement: Supplementary file 1 [file biology-12-00822-s001.zip › Figure S3.pdf]

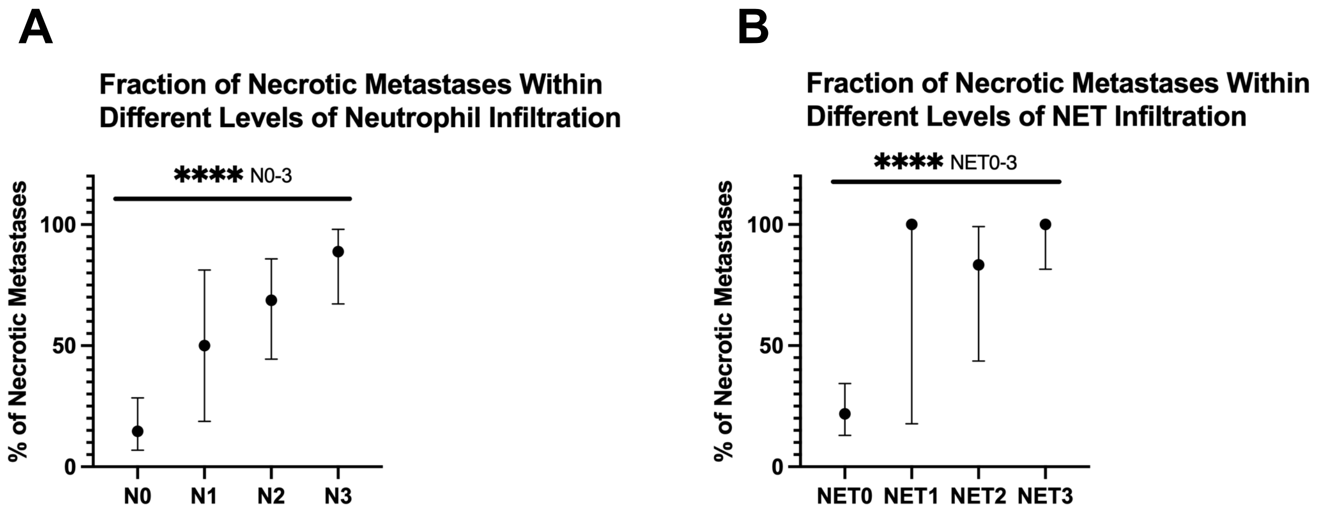

Figure S3. Chi Square analysis of necrosis and the different levels of neutrophil (A) and NET (B) infiltration revealed a strong association ($p<0.0001$ in both). Comparing the proportions of necrotic metastases between the different levels shows higher amounts of necrotic metastases in N2 and N3 or NET2 and NET3 compared to N0 or NET0, respectively.

Proportions are shown as percentages with 95% confidence interval.
