# Supplementary material for: Neutrophil Extracellular Traps Correlate with Tumor Necrosis and Size in Human Malignant Melanoma Metastases
Source: Biology (Basel). 2023 Jun 6;12(6):822. doi: 10.3390/biology12060822 (PMC10295294; doi:10.3390/biology12060822)
Supplement: Supplementary file 1 [file biology-12-00822-s001.zip › Figure S4.pdf]

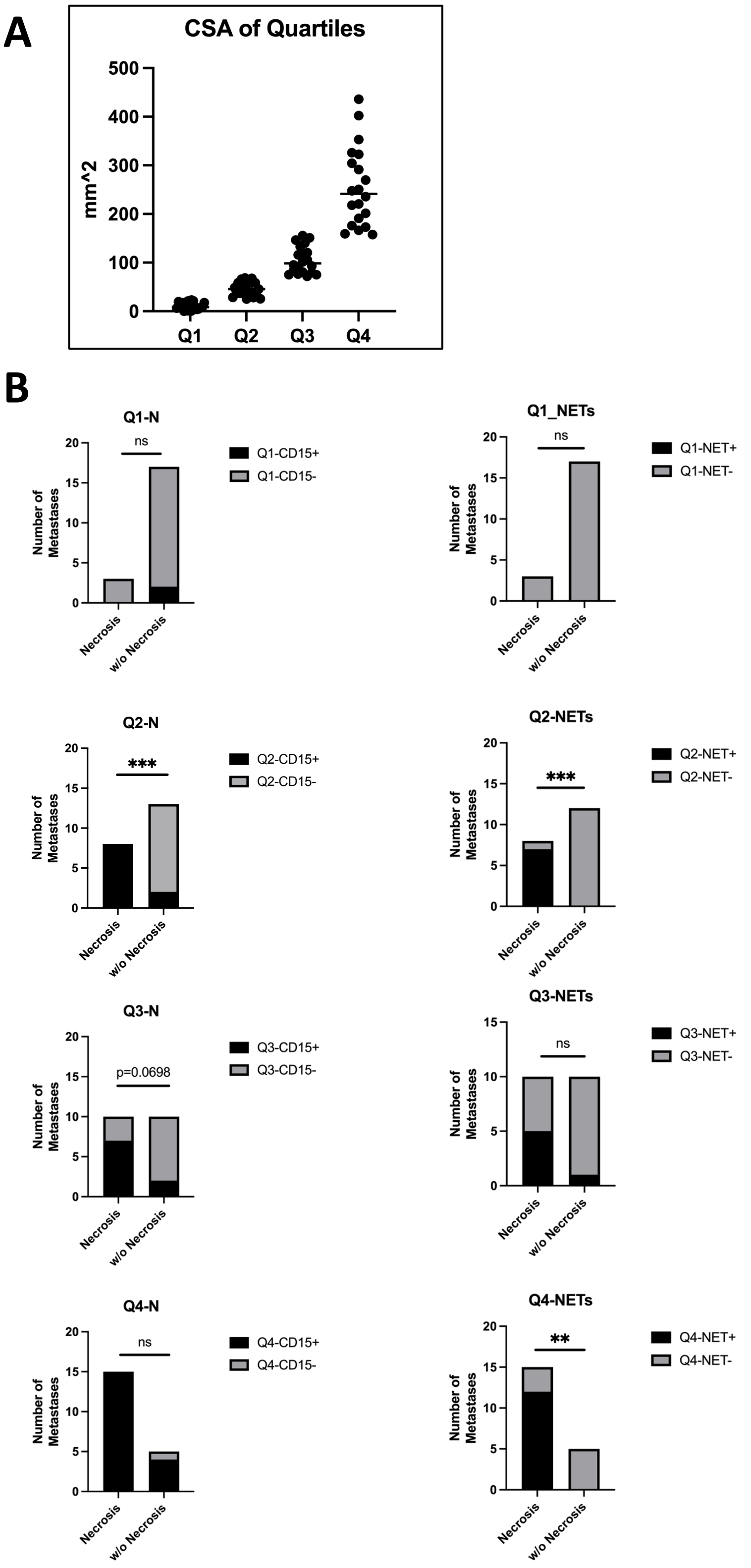

Figure S4. Analysis of neutrophil and NET infiltration in Quartiles.
A Overview of Quartiles (with median).
B Results of comparison within Q1-Q4.
