# Supplementary material for: Neutrophil Extracellular Traps Correlate with Tumor Necrosis and Size in Human Malignant Melanoma Metastases
Source: Biology (Basel). 2023 Jun 6;12(6):822. doi: 10.3390/biology12060822 (PMC10295294; doi:10.3390/biology12060822)
Supplement: Supplementary file 1 [file biology-12-00822-s001.zip › Table S1.pdf]

Table S1 Overview of analyzed metastases

Pat.	Metastasis	Localisation	CSA	max Diamete	Necrosis	Neutrophils	NETs	Neutrophils	NET
			in mm^2	in mm	1=yes; 0=no	1=yes; 0=no	1=yes; 0=no	N (0-3)	NET (0-3)
A	1	Lungs	7,18	n.d.	0	0	0	0	0
B	2	Skin	6,86	n.d.	0	0	0	0	0
B	3	Skin	28,62	10,00	0	0	0	0	0
C	4	Skin	68,57	n.d.	0	0	0	0	0
D	5	Skin	150,96	80,00	1	1	1	3	3
E	6	Lymph Node	58,42	10,00	0	0	0	0	0
F	7	Lymph Node	201,39	n.d.	0	0	0	0	0
G	8	Lungs	9,37	n.d.	1	0	0	0	0
H	9	Lymph Node	21,41	n.d.	0	0	0	0	0
I	10	Skin	92,00	n.d.	0	1	1	3	2
J	11	Skin	75,13	n.d.	1	1	1	2	1
K	12	Skin	21,01	n.d.	0	0	0	0	0
K	13	Skin	218,32	n.d.	1	1	1	3	3
K	14	Skin	83,26	12,00	1	0	0	0	0
L	15	Lymph Node	80,61	10,00	0	0	0	0	0
M	16	Skin	22,92	n.d.	0	0	0	0	0
N	17	Skin	17,50	7,00	0	0	0	0	0
O	18	Skin	45,83	n.d.	0	1	n.e.	1	n.e.
P	19	Skin	36,69	10,00	1	1	1	2	2
Q	20	Lymph Node	79,26	10	0	1	0	3	0
R	21	Lymph Node	166,34	26	1	1	0	1	0
R	22	Skin	6,64	6,00	0	0	0	0	0
R	23	Lymph Node	8,09	6,00	0	1	0	2	0
S	24	Lymph Node	436,02	n.d.	1	1	1	3	3
S	25	Lungs	19,93	n.d.	1	0	0	0	0
S	26	Unclear (Skin	402,47	70,00	1	1	1	3	3
T	27	Lymph Node	116,74	13,00	1	0	0	0	0
T	28	Lymph Node	101,52	12,00	0	0	0	0	0
U	29	Skin	31,18	8,00	1	1	1	1	1
V	30	Skin	159,27	n.d.	1	1	1	2	3
W	31	Skin	45,71	n.d.	1	1	1	2	3
W	32	Lungs	140,32	n.d.	1	1	0	3	0
X	33	Skin	22,01	7,00	0	1	0	1	0
Y	34	Skin	47,83	n.d.	0	1	0	1	0
Z	35	Skin	76,38	n.d.	0	0	0	0	0
Z	36	Lymph Node	326,14	n.d.	1	1	1	3	3
Z	37	Lymph Node	157,52	15,00	1	1	0	2	0
Z	38	Skin	38,13	6,00	0	0	0	0	0
AA	39	Skin	17,41	7,00	1	0	0	0	0
AA	40	Skin	173,17	44,00	1	1	1	2	2
AA	41	Lymph Node	27,99	8,00	1	1	1	1	3
BB	42	Skin	48,70	7,00	0	0	0	0	0
CC	43	Skin	41,83	9,00	1	1	1	3	3
CC	44	Skin	95,19	n.d.	0	0	0	0	0
DD	45	Lymph Node	120,83	n.d.	0	0	0	0	0
EE	46	Lymph Node	25,77	6,00	0	0	0	0	0
FF	47	Skin	0,04	8,00	0	0	0	0	0
GG	48	Lungs	17,77	6,00	0	0	0	0	0
HH	49	Lungs	7,32	n.d.	0	0	0	0	0
II	50	Lymph Node	1,23	n.d.	0	0	0	0	0
II	51	Lymph Node	71,72	10,00	1	1	1	3	3
JJ	52	Lymph Node	37,80	n.d.	0	0	0	0	0
KK	53	Lymph Node	25,34	18,00	1	1	1	2	3
LL	54	Lymph Node	105,71	12,00	0	0	0	0	0
MM	55	Lymph Node	191,21	n.d.	0	1	0	2	0
NN	56	Lymph Node	132,94	n.d.	0	0	0	0	0
OO	57	Lymph Node	0,74	6,00	0	0	0	0	0
PP	58	Lungs	75,15	n.d.	1	1	1	3	2
QQ	59	Skin*	269,59	n.d.	1	1	1	3	3
RR	60	Liver	235,44	n.d.	0	1	0	2	0
SS	61	Lymph Node	291,31	n.d.	1	1	1	3	3
TT	62	Lungs	58,59	n.d.	0	0	0	0	0
UU	63	Lungs	304,24	n.d.	0	1	0	2	0

VV	64	Liver	3,75	1,50	0	0	0	0	0
VV	65	Liver	322,68	n.d.	1	1	1	3	3
WW	66	Skin	155,63	n.d.	1	0	0	0	0
XX	67	Unclear (Parc	220,55	n.d.	1	1	1	2	2
YY	68	Liver	175,85	n.d.	1	1	0	2	0
ZZ	69	Liver	65,09	n.d.	1	1	1	3	3
ZZ	70	Liver	250,61	n.d.	1	1	1	3	3
AAA	71	Liver	247,71	n.d.	1	1	1	2	2
BBB	72	Liver	58,04	n.d.	1	1	0	3	0
BBB	73	Liver	353,14	n.d.	0	1	0	2	0
CCC	74	Liver	93,32	n.d.	0	0	0	0	0
DDD	75	Liver	146,40	n.d.	1	1	0	2	0
EEE	76	Liver	57,75	n.d.	0	0	0	0	0
FFF	77	Liver	115,95	n.d.	1	1	1	3	3
GGG	78	Lungs	4,83	n.d.	0	0	0	0	0
HHH	79	Lungs	14,63	n.d.	0	0	0	0	0
HHH	80	Lymph Node	66,00	n.d.	0	0	0	0	0
HHH	81	Lymph Node	67,60	n.d.	0	0	0	0	0

Explanations:
n.d. = not documented (in pathological record)
n.e. = not evaluable
* = ulcerated
